# Contributions to Obstetric Jurisprudence

**Published:** 1859-07

**Authors:** Horatio R. Storer

**Affiliations:** Boston


ART. I.—*Contributions to Obstetric Jurisprudence.* By HORATIO R. STORER, M.D., of Boston.

### CRIMINAL ABORTION. (CONTINUED.)

### VI. ITS INNOCENT ABETTORS.

WE have referred to an apparent disregard of fœtal life, obtaining in the medical profession, as a prominent cause of the prevalence of criminal abortion. We now proceed to show that the opinion is not unfounded.

Premature labor, or obstetric abortion, may be justifiably induced by the physician for one of two reasons; either to save the life of the mother or that of her child. In each case it must be, absolutely and only, to save a life.

Performed for the child's sake, it is evident that the operation can be only available during the last three months of pregnancy, for then only can the fœtus with any degree of probability be considered viable. We grant that there are a few cases on record where, born during the sixth month and even in the fifth, the child has survived; but it is equally certain, despite the popular notion concerning the mortality of eighth-month children, that the later the operation can be safely delayed, the better the chance for the infant's life.

The rules for the induction of premature labor must, of course, vary for different cases. In this early stage of the inquiry, it is perhaps impossible to state them precisely, but they may still be approximately arrived at.

1. The operation, performed for the child's sake, is but seldom required; and in general,

(2.) only after the commencement of the seventh month of pregnancy.

3. It must be clearly indicated; and

(4.) must be delayed as long as is consistent with the child's safety.

5. Its means must be those which are most efficient, and safest for the child.

We have already stated that the induction of abortion before the seventh month, undertaken for the child's sake, must be generally useless; and therefore, as attended with some degree of danger to the mother, generally unjustifiable. As the profession are nearly united on this point, its further discussion is here unnecessary.

We have asserted that the cases where prematurely induced labor is required for the child, are comparatively rare. We now add that while in some respects they are more frequent, in others they are less so than is generally supposed. To necessitate it, there must be disease or deformity on the part of the mother, or disease on the part of the fœtus or its appendages.

It is most frequently performed to avoid the alternative of craniotomy, the necessity of which, unless extreme, can manifestly only be known with certainty, before the expiration of pregnancy, from the experience of past labors. But here too much caution cannot be exercised; the rules of the books and of accepted authorities are not to be blindly followed.

Craniotomy at the full time is still too frequently performed:

Where it has been suggested by the character of a previous labor, children are often, or might be, born living;

Where it seems indicated by direct exploration, as ruled even by recent writers, children are sometimes, or might be, born living;

Where it was formerly thought absolutely essential, the progress of obstetric science has now rendered it often unnecessary.

It is proper that we consider these points, for they bear directly on the question at issue concerning criminal abortion.

It is known that the sex of children exercises an appreciable influence upon the result of labors at the full time, as regards the possibility of their passing alive, unaided or at all, through the pelvis, and as regards the length of the labor, which also progressively endangers the life of the mother and their own; the average female fœtus, in cases at all difficult, having the advantage over the male by its inferiority in size, especially important in the cranial diameters. It is unnecessary for us to do more than refer to the facts by which these assumptions are proved.* Where, therefore, craniotomy has been found necessary in a former labor, the child then being male, in another labor a female fœtus may often pass uninjured. However this argument may be lessened in value by the impossibility of previously ascertaining the sex, it is strengthened in the doctrine of chances, by the number of the labor and the sex of the former children; and by the fact that first labors are generally most difficult, whatever the sex of the child.

Furthermore, cases will suggest themselves to most practitioners of experience, in which from difference in the character of the labor or without apparent reason, children are born living at the full time, males, of large size, and presenting by the vertex, where craniotomy had previously, perhaps repeatedly, been performed. An instance of this has occurred to

---

* SIMPSON, Obst. Works, i. pp. 352, 404.

the writer, where the patient had been advised to early abortions from alleged physical incapacity of ever bearing living children; he delivered her, without difficulty, of a large-sized and living boy, at the full time.

Again, it may happen that in labor at the full period, craniotomy may seem decidedly indicated and advisable, but for one reason or another may not be performed, and yet the child, unaided, be born living. The writer's experience furnishes him with two illustrations of this class: one of them occurred in 1851, the patient his own, Drs. S. sen., and C., in consultation; the other was seen at Edinburgh, in 1854, in the practice of his friend, Dr. G. W. In both cases, permission was refused to operative procedures, the patients being Catholic, and the physicians unable conscientiously to pronounce the fœtus already dead, while the possibility of intra-uterine baptism was not recollected; in both instances the children were born living, and without instrumental aid.

In other cases, where from deformity previously diagnosticated, craniotomy is pronounced necessary if the patient should go her full time, she may do so, the labor be unassisted, and the child yet escape with its life. I instance a remarkable case for some time under my own charge at the Boston Lying-in Hospital, delivered by one of my colleagues, Dr. Dupee, and subsequently reported by Dr. Read.* The pelvis was here equably contracted, and to a great degree, but from some difference of opinion, partly as regarded the justifiability of premature labor as compared with craniotomy, the patient was allowed to go her full time. Both mother and child did well.

Finally, by turning, the use of the long forceps and of anæsthesia, children are now constantly saved, where formerly craniotomy and their consequent destruction would have been absolutely indicated. These processes, with the introduction of each of which as an alternative a single name is imperishably connected, are now successfully employed by very many of the profession;† they have each of them saved to the writer the disagreeable necessity of fœtal destruction. Where, however, one life is thus preserved, there are still multitudes unnecessarily, and, therefore, unjustifiably, sacrificed.

The comparative frequency of craniotomy in the different countries of Europe is in this connection worth noticing.

---

* Boston Med. and Surg. Journal, January, 1857, p. 462.

† The immorality of craniotomy, where delivery can be effected by any other method, is gradually becoming acknowledged in Great Britain. A late discussion on this subject, at the Obstetric Society of London, is reported in the Medical Times and Gazette for February, 1859.

The operation is performed, in

| | | | |
|---|---|---|---|
| Germany . . . . . . | once in every | 1944 | labors; |
| Paris . . . . . . . | " " | 1628 | labors; |
| France, at large . . . . | " " | 1200 | labors; |
| Vienna . . . . . . | " " | 688 | labors; |
| England . . . . . . | " " | 220 | labors; |
| Ireland, formerly . . . . . | " " | 128 | labors;* |
| " at present, Dublin Hospital, 1854, | " " | 105·7 | labors.† |

The remarkable difference between the practice on the continent and in England, so suggestive to us in this country, is undoubtedly owing to the fact that in Catholic States greater value is attached to the life of the child than "in Protestant States, as Britain, where the child is always sacrificed to save the mother."‡ The immense excess of embryotomy cases in Catholic Dublin furnishes no exception to this rule, drawn as they are from hospital practice under Protestant control.

So far proof by deduction. In many cases involving the question of craniotomy, that operation is not required; in some of them, not even the induction of premature labor.

Two classes of cases remain, each affording more direct evidence; those where craniotomy being absolutely indicated if the patient were allowed to go her full time, that operation is, and those where it is not performed.

Craniotomy, being necessarily fatal to the fœtus, is indicated only to save the mother's life; to be avoided when any other alternative giving the fœtus a chance of life, and not more than equally hazardous to the mother, can be resorted to. Especially is this the case when it is compared with the induction of premature labor, attended as is the latter, despite a certain amount of danger of its own, with great probability of saving the child, and with decidedly lessened risk to the mother; for craniotomy not merely requires the use of murderous instruments, dangerous to all tissues they may approach or be in contact with, but the operation is usually, though often very improperly, delayed till late in labor, and therefore till the mother's chances of recovery have been proportionally lessened. Premature labor, on the other hand, though of course involving some risk to the child, is not necessarily fatal to it; nor is it usually so, when properly performed. That there is a choice in this respect between the means employed, will hereafter be shown.

---

* CLAY, Obstetric Surgery, p. 68.

† SINCLAIR and JOHNSTON, Practical Midwifery, 1858. 130 cases of craniotomy in 13,748 labors.

‡ CLAY, Loc. cit., p. 69.

Craniotomy, when absolutely indicated at the close of pregnancy, must be for one of two reasons: that the fœtus cannot pass through the pelvis at the full time alive, though it may do so unmutilated, the operation being performed to save the mother the greater risks of protracted labor; or that it cannot pass at the full time unmutilated, even when dead. We defer the consideration of another supposable instance, where the fœtus in the outset of its viability may pass, but not alive, for this pertains to the consideration of the mother's safety alone, no alternative availing for the child. In the other cases, if the necessity could be learned in season, from the previous history of the patient or by pelvic exploration, labor should most certainly be prematurely induced, as affording some positive chance of life to the child, and as less dangerous to the mother. It were here worse than foolish, if not criminal, blindly to imitate nature, when, her course being obstructed, she would kill the child.

I have alluded for a double reason to cases, fortunately few, where craniotomy, and much more decidedly premature labor being indicated, the practitioner decides from the outset to perform neither, to give his patient or her child no aid. Such conduct is as cruel and wicked as it is unprofessional, and were not instances occasionally reported, its existence could hardly be believed. We acknowledge with Blundell the evils of meddlesome midwifery, but there are extremes to all things; certainly to the powers of nature and the limits of justifiable delay.

I am aware that I have referred to a reported case, which might in this connection be quoted against the opinion now expressed; but even by its exceptions do we prove the rule. Where chances are so greatly against both mother and child as in these cases if left unaided, it would be the office of the physician, were there no better procedure, by craniotomy to save the one;* but at the present day it is no less plainly his duty, where possible, to anticipate labor, and thus save both. I have elsewhere discussed this question at some length,† and can only repeat, as is indeed allowed by Churchill,‡ that this is no matter on which to select one's

---

* The subject of justifiable craniotomy has of late been ably though controversially discussed by an anonymous writer (Dublin Review, April and October, 1858,) and Dr. Churchill (Dublin Quarterly Journal of Medical Science, August and November, of the same year.) Care must be taken, lest in assenting to the decided and imperative necessity of the operation in certain cases, and by a natural professional sympathy, too great frequency is not allowed to this most horrible and appalling of all the operations to which as physicians we can ever be called.

† Review of Clay's Obstetric Surgery; Boston Med. and Surg. Journal, November, 1856, p. 283.

‡ Theory and Practice of Midwifery, p. 348.

words; the deliberately sacrificing an unborn, but still living child, in cases where statistics go to prove that the adoption of another mode of delivery, nothing counter-indicating, would give that child a good chance of successful birth, is nothing short of *wilful murder*, no matter by what schools or by what eminent men it may be sanctioned, and it should be branded as such by the profession.

But, undertaken for the child's sake, not merely should premature labor be resorted to for the purpose of preventing craniotomy, but often in cases of incurable disease, acute and chronic, of the mother, where it is evident that she must inevitably, or even probably, perish before the full period of pregnancy has been attained. Instances of such acute disease will readily suggest themselves. The question here is merely between the operation under consideration, the Cæsarean section, and doing nothing. The last, suppose the fœtus viable and to be still living, would in many instances be decidedly unprofessional and unjustifiable. Cæsarean section after the mother's death is comparatively unsuccessful; and before it, is so much more severe and in all probability so much more quickly fatal, that the other should be preferred, unless death be already close at hand.

Instances of chronic incurable disease necessitating the induction of premature labor, may not so readily occur to the mind. The shock of an abortion being frequently greater to the maternal system than that of labor at the full time, it is evident that this rule cannot be universally applied. It cannot in every case of thoracic disease, of the heart, for example, unless its own peculiar symptoms become so aggravated from progressing pregnancy as to render probable earlier decease of the mother, the operation then being performed partly on her account; not so much to save her life as to delay a little her death. But there are other and extreme cases, as cancer of the lower segment of the uterus, or indeed of its fundus, which may have been diagnosticated previously to pregnancy by the use of expansible tents, or of my uterine dilator hereafter referred to, where the mother would probably perish in labor at the full period, with most probably the loss of the child also. Here, by premature labor, the child may be saved, and the mother's life, greater expansion of the uterus and its more probable laceration being prevented, possibly prolonged.

So far complications on the part of the mother necessitating premature labor for the sake of the child. There are others equally imperative, afforded by itself. Excessive size of the fœtus in comparison with a normal pelvis, as evidenced and rendered probable by previous labors, is hardly of less importance in connection with craniotomy, than where the pelvis is distorted or contracted, and the fœtus of natural size; but the operation in question, as compared with premature labor, we have already sufficiently discussed.

Diseases of the placenta, congestive, inflammatory or degenerative, are no less an indication, where known to exist, for an early delivery. Their diagnosis may be difficult, but yet not wholly impossible. The occurrence of the same disease in past labors, and evident intra-uterine disturbance as discovered by auscultation or by unnaturally frequent and strong fœtal movements, are, taken together, frequently sufficient to establish the fact. The impropriety of allowing such cases to proceed unaided, cannot be too strongly insisted upon. By early delivery, if the fœtus have formerly perished after the period of viability, and in addition, by special medication of the parent, if its death had usually occurred before that time, many valuable lives might annually be saved.

Premature labor as resorted to on the mother's behalf alone, putting aside the cases we have incidentally considered, where the lives of both herself and her offspring are of necessity taken into account, includes also its induction in the earlier months of pregnancy before the fœtus is viable. It may be required by diagnosticated malformation or monstrosity of the fœtus and by malformations on the part of the mother; by extreme pelvic contraction, congenital or from rickets or malacosteon, preventing the natural passage of a fœtus after viability, by tumors incapable of elevation or displacement, by contraction of the vagina, or by other severe obstetric complications, either recent or of long standing. Of this last class of causes, obstinate vomiting, puerperal convulsions, which are by no means confined to the full period of pregnancy, dropsies, irreducible displacements of the uterus, varix of the external labial vessels, sometimes fatal by laceration from tension, are all instances in point. With regard to each, the necessity of the abortion must be determined with the greatest caution, and resort be had to it, the child still living, with reluctance; especially should this be the case if the complication be supposed on the part of the fœtus, so important is it to avoid its sacrifice, if possible, and the semblance of disregarding its own important claims.

The rules for the operation when performed for the mother are the same as when for the child, save that if absolutely required, it may be resorted to at an earlier period. If the child must necessarily be lost, the labor should not be long delayed.

There are several subordinate questions arising in this connection, neither metaphysical nor merely casuistic, but practical, and because bearing on the increase of criminal abortion, directly involving human life to an indefinite extent. One of them we shall now mention.

Let physical incapacity to the birth of a living or viable child through the natural passages be supposed to exist on the part of the mother; that this has been proved by examination, or by the result of a former labor, induced or at the full time. To save the mother's life, early abortion is once brought on and the child destroyed; the woman and her husband being of course informed of the true state of the case. Is it right or justifiable, again to destroy the fœtus, and as often as sexual lust may repeat impregnation? Or should the patient be left to the risks of a subsequent Cæsarean section, which would at least give the chance of life to her child?

I am aware on which side of this question lies at present the opinion and the practice of the mass of the profession; that Nægele* and others† have ruled it right always to destroy the fœtus when a refusal to undergo the Cæsarean operation shall have been formally expressed by the mother; and that reports of instances where early abortion has been repeated, even to nine times upon the same patient, are still unblushingly published by men of the standing of Lever and Oldham.‡

But, on the other hand, in his late admirable justification of craniotomy where absolutely necessitated, Churchill makes use of the following language, which though offered in another connection, is none the less pertinent here: "It is the due appreciation of these relative responsibilities (regarding mother and child) in difficult cases, that distinguishes the wise and experienced accoucheur; he preserves a just counterpoise between them so long as it is possible to fulfil both, and recognizes the proper moment when one ceases. *One*, I say, not *either;* for I protest against the notion that *we choose which of the two lives we shall save*, a notion as false in theory as it is in practice. *No man dare make such a choice*, for we have neither the necessary knowledge, nor the right, nor the authority, to decide which is the more important life and best worth preserving."§

"My own opinion is that such a course (the repetition of abortion) ought not to be adopted, but that pregnancy should be allowed to proceed, without interruption, to the full period; and when labor declares itself, that the infant should invariably be extracted by the Cæsarean section,"|| which, when performed in season, is by no means necessarily fatal to the mother, and may preserve life to the child.

---

* De jure vitæ et necis quod competit medico in partu. Heidelberg, 1826.

† "Where one only can by any possibility be preserved, the female herself may use her right of self-preservation and choose whether her own life or that of her child shall fall a sacrifice." GUY, Principles of Forensic Medicine, p. 145.

‡ Guy's Hospital Reports, 1856, p. 12.

§ Dublin Quarterly Journ. of Med. Science, August, 1858, p. 10.

|| RADFORD, British Record of Obst. Medicine, 1848, p. 84.

The question now so plainly put, is one for the profession soberly to discuss and to answer.

We have already shown that in many cases where instrumental delivery or the induction of premature labor is apparently requisite, the mother, if a Catholic, is sacrificed to the supposed impossibility of administering the right of baptism to the child. We do not, with some, allow that the physician is here justified in deliberately and falsely asserting the child's death, where such has not taken place, but we have revived the suggestion of a method by which this great and fundamental obstacle may be overcome. We assert that the negligence too often shown by physicians in these cases, the custom of practically leaving the mother and her child to their fate when instrumental delivery shall have been refused upon religious grounds sincerely entertained,* is, however wide it may appear from the point, directly incentive to the increase of criminal abortion. Provided the question of necessity is determined, but one course in these cases should be pursued, and that the performance of intra-uterine baptism.

The objections usually made to the induction of premature labor, namely, the uncertainty of all pelvic measurements and of the exact period of gestation, the greater liability to malpresentation, and, from the uninvoluted state of the cervix, to the evils of a lingering labor, lose much of their force when tested by our preceding remarks. If resorted to, the means of its induction are various, and, as regards their justifiability, they present a decided choice. We here omit the consideration of the methods indirect or of doubtful efficacy, as draughts, general or local baths or bleedings, forced exercise, fatigue, and voluntary falls or blows, so frequently resorted to by the uneducated, or used, in addition to other procedures, by the designing to mask the reality; these will be subsequently considered, and we confine ourselves, in comparing the other and more direct methods, only to ascertaining that which is safest and most efficacious, for these points alone can decide their respective justifiability.

The direct and reliable means of inducing abortion, in the physician's possession, are only instrumental. Draughts of all kinds, whether purgative, emmenagogue, or so-called specific,—aloes, ergot, or savin,—or energetic poisons, expelling the fœtus through a sudden and profound disturb-

* The rules of the Catholic Church upon this point have been already referred to. Suffice it to say, further, that while they enjoin the Cæsarean and vaginal sections, in preference to craniotomy and in cases of extra-uterine fœtation, yet turning, the use of forceps, and the induction of premature labor, where such are indicated, are distinctly allowed by them. BARRY, Medico-Christian Embryology, pp. 41, 44, 45, 60.

ance of the whole maternal system, as arsenic or cantharides, are too unreliable, unscientific, or dangerous either to mother or child. The justifiable methods are confined to those acting directly on the uterus and its contents, by dilating the os or detaching the membranes.

They are, rejecting the local application of belladonna as utterly inert:

1. Local or distant and sympathetic irritation
   By the hand;
   medicinal applications;
   electricity or galvanism.
2. Puncture of the membranes, and evacuation of their fluid contents.
3. Dilatation of the os, and separation of the membranes from the uterine walls
   By sponge tents;
   the introduction of instruments;
   or the injection of fluids.

The first of these modes, in all of its applications, is extremely uncertain in effect.

The second, though that usually attempted, is of real avail only toward the close of pregnancy, and is even then decidedly inferior to the last of the methods proposed; at an earlier period it is probably unjustifiable. In thirteen cases reported by Lever and Oldham,* where labor was prematurely induced by puncturing the membranes, only four of the children were born living, and in all these the presentation was by the vertex. It has been shown that malpositions are much more frequent in earlier than later pregnancy, and from evident causes, the comparative absence and weakness of the fœtal movements which govern its position in utero, and the want of correspondence in form between itself and the containing organ. Of the nine cases reported where the fœtus was dead, five presented by the vertex, and were therefore probably alive at the commencement of labor, as after death previous to labor, malposition very generally occurs, except there be deficiency of the liquor amnii or the pregnancy have advanced to near its close.

Again, it appears from the same statistics, which as offered for another purpose are the more to be depended upon, that in the successful cases, where the child was born living, the length of time after the operation and before the occurrence of pains, almost doubled that in the unsuccessful cases, where the child was lost; the sum of the hours in the four successful cases exceeding the sum in the nine unsuccessful ones. That is to say, in the one class of cases time was allowed for a certain amount of

* Guy's Hosp. Reports, 1856, p. 4.

dilatation of the os and cervix and for some degree of detachment of the membranes, an approach, however slight, to the characters of normal labor; and in the other cases the labor was brought on at once, without either of the above processes having commenced, and when both uterus and its contents were totally unprepared. Had the times, moreover, from the commencement of labor to its close, been given in the two classes of cases, it would probably have been found that the relative proportions were reversed; that the sum of the hours in the unsuccessful cases exceeded that in the successful cases, as would be in strict accordance with the results to the fœtus.

In premature labor, as in that at the full period, the bag of waters is required to be preserved until it has fulfilled its purpose, on the one hand, of a fluid wedge, and on the other, of protection to the child from violence till it has fairly entered the pelvic brim. Puncture is also attended with the danger of directly wounding the uterine tissues.

The last of the methods referred to, dilatation and detachment, combined to a certain extent as they must necessarily be, is in close imitation of the processes observed in natural labor, and is recommended alike by its safety to mother and child, its certainty, and the ease both to physician and patient with which it may be effected.*

From the above remarks it is evident that the profession need to exercise great caution lest they directly, though unintentionally, become the abettors of criminal abortion. But this liability is not confined to the instances already mentioned, where abortion has been intentionally induced. Nor is the matter in question one affecting merely the public health and morals, like prostitution or syphilization. It is a liability directly to increase the unjustifiable destruction of human life, and its existence cannot be too strongly impressed upon our minds and guarded against.

So true is this, that two of the French obstetricians, Moreau and Bégin, have not hesitated to express their fears, and not on religious grounds alone; the latter, with Tardieu, giving it as his conviction, that every

---

* For a full discussion of the respective merits of the several methods instanced above, see SIMPSON, loc. cit., i. p. 738.

I have lately contrived an instrument very similar to one not long since proposed by Spencer Wells for dilatation of the female urethra, which by a simple combination of the three principles involved, will probably prove of material service in the induction of premature labor. It may be called the uterine dilator, as it possesses many advantages over expansible tents for all cases of uterine disease where dilatation is necessary, either for diagnosis or treatment. A description of the instrument, and of its first application to obstetric practice, is published in the current number of the American Journal of the Medical Sciences.

physician should make a legal declaration of the act to the public prosecutor, immediately on inducing premature labor, even if the period of viability has been reached by the fœtus. The subject has also been discussed by the French Academy of Medicine;* and in Great Britain it has been referred to by Radford, in whose opinion an enactment is necessary, "entirely prohibiting obstetric abortion (before the period of viability), as the door for evil purposes is already too open, and would be still more so, if it was legally decided that where performed on supposed obstetric grounds, no inquiry should be made."†

Whenever an abortion is induced by a physician, even if accidentally, it is liable to be thought intentional by the patient and her friends, and consequently by the community, so far as it becomes aware of the fact. Instances of this are within the writer's knowledge.

Accidental abortions, caused by a physician, may be from two causes: error or insufficient care in diagnosis, or the absence of all likelihood of the existence of pregnancy. When cases are reported in apparent innocence of fault, and by eminent practitioners, of the pregnant uterus having been tapped for ovarian dropsy; of craniotomy having been attempted upon an infant's fundament instead of its skull, and of the same operation in another instance undertaken upon the promontory of the mother's sacrum, it cannot be alleged, I care not by whom, that this caution is unnecessary or these fears unfounded. The writer will state a few cases that have happened under his own observation. He has twice known abortion to be accidentally occasioned by the use of sponge tents;‡ the patients being near the close of their menstrual lives, and neither themselves nor the physicians in attendance supposing that the contents of the enlarged uterus could be fœtal; in one of these cases the woman had never before been pregnant, and in the other not for many years. He has twice known the introduction of an intra-uterine pessary to be followed by abortion; in neither case was there any probability of the existence of pregnancy, save in the fact that the patients were married. He has known abortion to be produced by the application of lunar caustic to the os, in a case where, from the character of the operator, no suspicion could be entertained of the uterus having been more deeply tampered with.

In cases like these, the practitioner would seem to render himself liable to the charge of malpractice, even though by the improbability of malicious intent he escaped that of criminal abortion. In all instances where there exists the slightest suspicion or possibility even of pregnancy, the

---

* Bulletin de l'Académie, xvii. p. 364.

† British Record, etc., p. 82.

‡ I have elsewhere discussed this subject; American Journal of the Medical Sciences, January, 1859.

only rule for operative interference must be, unless circumstances imperatively prevent, to wait the few months or weeks necessary to establish the diagnosis. Justice to the profession and to the risk of error demand that where such precaution is not had, it should be replaced by a consultation, which would at once dispel any suspicion of carelessness or malpractice, and prevent a criminal charge.

All that we have said regarding surgical obstetrics, applies as forcibly to its medicinal procedures. Though draughts and potions of every kind are, as we have remarked, of doubtful or indirect efficacy for inducing abortion, yet with some of them instances of its occurrence do at times result. In supposed obstructive amenorrhœa, unless of several months standing, and in early pregnancy where established, the physician who does not intend it, must take care lest accidentally or carelessly, by purging, vomiting or over-bleeding, he produce a miscarriage.

But there is still another point in this connection we must not pass over. We state it in the following inquiries, received from one of the most eminent practitioners of the Eastern States, and put to us in all sincerity.

"Are there not cases where a physician would be justified in suggesting a course such as he would use in amenorrhœa, even where he might *suspect*, but not *know*, the existence of pregnancy? Suppose a mother of several children, which she has had in rapid succession, and the physician feels assured that health, and possibly life, will be endangered if another pregnancy occurs; would he be criminal if he were to use common means for amenorrhœa if the menses have been absent six weeks? Are the cases always so plain that a man can decide, and may he not balance a choice of evils?"

Covering as these questions do, much of the ground already gone over, we may answer them at once, and decidedly in the negative. They apply more especially to the early months of pregnancy, where it is always impossible to know its existence; and were direct or probable emmenagogues, or instrumental interference, to be here allowed, criminal abortion might be always induced before quickening, sanctioned and permitted by the rules of medicine. To justify abortion, life must certainly or very probably be endangered, not possibly merely, which is true in every pregnancy, and might be alleged at every trial for the crime.

Hufeland advised, as a "golden rule," always to suppose the existence of impregnation in such cases, and to act accordingly; that is, to temporize long enongh for the fœtus, if present, to make its sign. "Thereby," he says, "the physician will avoid much mischief, and preserve his conscience as well as his reputation."*

---

* Enchiridion Medicum, p. 510.

The ideas on this subject held by my friend, are without doubt widely entertained by the profession. This paper will have served a good purpose and have saved many fœtal lives, if it do no more than carry conviction of the error and its likelihood to indefinitely extend the crime.

Again, it is undoubtedly the case that in all cases of maternal death during pregnancy, where the fœtus has arrived at the period of viability, its immediate extraction by the Cæsarean section should be effected. We have already referred to this subject, and Kergaradec, who has well written upon it, lays down the maxim that the operation should always be performed, even in the fifth month of pregnancy.* In some cases, of placenta prævia, for instance, where the chances are always more or less against the child's life, success is less probable; but this in no wise invalidates the necessity of the operation. The writer has himself performed it in the complication instanced, and in vain; but he would none the more hesitate, on this account, to repeat it. There can be no reason against the procedure in any case, and by it the child may possibly be saved. In the words of an older writer, "Est enim inhumanum, post obitum matris, fœtui pereunti et suffocari parato manus auxiliares denegare, et sæpè viventem adhuc cum matre mortuâ eodem tumulo contegere et obruere. Idcirco jurisconsulti eum *necis* reum damnant, qui gravidam sepelierit non prius extracto fœtu."†

A question has been raised concerning the rights of relatives in preventing the physician from such discharge of his duty. It has been asserted that "the father has not only the natural right of his relationship, but legal power; for Dr. Lever recently mentioned that he had consulted Dr. Alfred Taylor to know whether he would be justified in performing Cæsarean section after the death of the mother, without the consent of the father, as it appeared unjustifiable homicide to allow the infant to die. Dr. Taylor gave his opinion that, in law, the infant belonged to the father,—the infant with the life thereof,—and that if Dr. Lever touched it, even to rescue it from death, an action would lie against him."‡ I must, however, declare such doctrine to be false and pernicious. If signs of the child's life remain, no physician should hesitate endeavoring to preserve it, unless restrained by actual force. I reiterate my conviction that such neglect, or seeming neglect, of fœtal life is an actual wrong, both against the individual and against society.

Similar points in which physicians are directly interested, as tending by

---

* Question d'Embryologie Médicale, etc.; Revue de l'Amorique et de l'Ouest, 1846.

† JOAN. RIOLAN., Anthropographia, lib. vi. cap. vii. p. 589.

‡ CHURCHILL, Dublin Quarterly Journ. of Med. Science, August, 1858, p. 22.

their apparent disregard of fœtal life to render themselves innocent abettors of criminal abortion, are not uncommon. Such are neglect of efforts to prevent miscarriage when threatening, or where it has become an established habit; and of attempts at resuscitating still-born children where there is the slightest chance of success, and success has now been rendered much more probable by the methods of Marshall Hall and Silvester; the performance of operations of any kind upon a pregnant woman, even tooth-drawing,* that might be delayed; the careless or unnecessary use of ergot; the relying upon a single and unaided opinion, where not one life only, but two, may be endangered.

Other instances might be adduced; but enough has already been said to prove that the importance of the subject we are considering, and the responsibilities resting upon the profession regarding it, demand as I have elsewhere suggested,† that physicians should possess, should acknowledge, and should govern themselves by, an *Obstetric Code*—the necessity of which will be made even more manifest, as we proceed in our investigation of questions pertaining to Obstetric Jurisprudence. We have referred to some of its leading principles, but have done no more than faintly foreshadow them.

Distressing in the retrospect, inconvenient frequently in the present,‡ such a Code would undoubtedly prove; but it is demanded of the profession by the progress of our science, by humanity, morality and religion. Were the facts in the case more generally known, and the existence and sanctity of fœtal life more universally appreciated, it would be also demanded by public opinion.

We have now seen that "the absurd enactments still remaining on the statute book, the careless indifference with which society views the crime, the reluctance with which means are adopted to prevent its occurrence, its increase, and its frequent induction by obstetricians, are all evils which loudly and imperatively call for the closest investigation."§

We proceed to the other relations of criminal abortion, more especially to those immediately pertaining to the claims and course of justice.

---

* A case in point has been reported by the writer; Amer. Journ. of the Med. Sciences, April, 1859.

† Reports to the Suffolk Dist. Med. Society of Massachusetts, 1857, and to the American Medical Association, 1859.

‡ "It would in my opinion," says Ramsbotham, referring to the nature of fœtal existence, "be much better not to endeavor to explain the secrets of nature, so deeply hidden."—(Obst. Medicine and Surgery, p. 309.) This belief seems still, in practice, very widely entertained.

§ CLAY, Obstetric Retrospect, March, 1848, p. 44.

(TO BE CONTINUED.)

